# Reliability and Validity of Inertial Sensor Assisted Reaction Time Measurement Tools among Healthy Young Adults

**DOI:** 10.3390/s22218555

**Published:** 2022-11-06

**Authors:** Brent Harper, Michael Shiraishi, Rahul Soangra

**Affiliations:** 1 Crean College of Health and Behavioral Sciences, Chapman University, Irvine, CA 92618, USA; 2 Fowler School of Engineering, Chapman University, Orange, CA 92866, USA

**Keywords:** reaction time (RT), wearable sensors, dual tasking

## Abstract

The assessment of movement reaction time (RT) as a sideline assessment is a valuable biomarker for mild TBI or concussion. However, such assessments require controlled laboratory environments, which may not be feasible for sideline testing during a game. Body-worn wearable devices are advantageous as being cost-effective, easy to don and use, wirelessly transmit data, and ensure unhindered movement performance. This study aimed to develop a Drop-stick Test System (DTS) with a wireless inertial sensor and confirm its reliability for different standing conditions (Foam versus No Foam) and task types (Single versus Dual), and postures (Standing versus sitting). Fourteen healthy young participants (seven females, seven males; age 24.7 ± 2.6 years) participated in this study. The participants were asked to catch a falling stick attached to the sensor during a drop test. Reaction Times (RTs) were calculated from data for each trial from DTS and laboratory camera system (gold standard). Intraclass correlation coefficients (ICC 3,k) were computed to determine inter-instrument reliability. The RT measurements from participants using the camera system and sensor-based DTS showed moderate to good inter-instrument reliability with an overall ICC of 0.82 (95% CI 0.78–0.85). Bland–Altman plots and 95% levels of agreement revealed a bias where the DTS underestimated RT by approximately 50 ms.

## 1. Introduction

Assessment of reaction times can provide valuable insights into mild traumatic brain injury (mTBI), often referred to as a concussion. Most reaction time studies use optoelectronic motion capture systems in laboratory environments [1]. Although these motion capture systems are highly accurate, they have limitations, including the requirement of a dedicated, controlled laboratory environment; bulky and expensive indoor equipment in a space large enough to take high-quality measurements; and complicated and time-intensive camera and video set-up for analysis.

Response or Reaction Time (RT) is defined as the time following the presentation of a stimulus during which perceptual and motor planning computations take place before the movement, and increased RT is commonly associated with neurological disorders [2,3,4,5,6]. It is feasible to measure response time which is a summation of perception time and movement time [2]. Response times vary depending on the kind of stimuli: visual, auditory, or tactile [7]. Experimental paradigms with RT have been used for decades to understand the information processing of the central nervous system to prepare and execute a response to a perceived stimulus [8,9,10]. A simple reaction time indicates the information processing speed [11,12]. For example, a fast reaction time reflects the intactness of the neuromuscular system [13]. A common cognitive sequela of concussion or mTBI is impairment in RT [14].

Decreasing the number and intensity of head impacts is essential to limiting the incidence of concussions. Several studies have found that athletes with faster RT sustained fewer and less severe impacts on the head [15,16,17,18]. It is theorized that faster RT increases an athlete’s anticipation of a hit, allowing them to protect their head from a more forceful blow [16,18]. Therefore, accurately and objectively quantifying visuomotor RT using metrics translatable from the laboratory to the clinic is paramount for creating a safe sporting environment.

Presently, objective dynamic visual-motor physical response options for on-field concussion testing are limited, reducing the return to play decision confidence due to a reliance on data with little objectivity. Commonly, laboratory-based visual reaction times are evaluated by recording the time it takes following the presentation of a light stimulus for a button to be pressed [19]. Despite the utility of these lab-based assessment metrics, there are no practical accepted measures of RT that have been validated in the lab and are translatable to on-field assessment use during an athletic event. Therefore, a simple, portable, and inexpensive method of measuring RT is needed in the training room and on the sideline for concussion assessment.

New wearable technologies such as inertial sensors and smartphones have provided many integrated ways to quantify human movement in out-of-lab environments [20,21]. These sensors allow subcomponents of any activity to be measured and analyzed. Some of these technologies have been proven reliable for gait and postural measures [22,23]. One easily quantifiable RT test used in previous research involves the stick drop RT test, in which an unexpectedly dropped object is caught by a participant [24]. In one drop test, the participant was instructed to stop the descent of a stick by pressing it against a wall with their thumb as soon as possible after seeing it dropped by the examiner. The RT was represented by the distance the stick fell before being stopped by the participant [24]. Such tests are easy to use on-field but require a wall or support surface.

Eckner et al. (2009) proposed a more clinical RT test in which a participant was instructed to catch a weighted stick which was dropped in random intervals of 4–15 s [25]. RT time was measured by recording the distance the stick fell before it was caught and the time it took to catch it. This drop-stick test system (DTS) could identify concussed athletes with a sensitivity of 75% and specificity of 68% [26]. This system was reliable to standard computer-based RT tests and was correlated with an increased head protective response [17,27].

Eckner and coworkers assessed the stick drop test using an accelerometer during a dual-task activity in which the athletes caught the stick only if a green LCD light was illuminated [28]. However, the reliability of the accelerometers to obtain this information has not been compared with other quantifiable metrics, nor was the dual task performed on any other surface or position. Furthermore, MacDonalds and coworkers questioned the reliability and validity of the DTS compared with computer-generated RT measures. They recommended further testing before incorporating such visuomotor RT tests into the clinic [29].

Since RT appears to be a valuable identification metric to assess individuals who may have sustained a concussive event, it is clinically significant to have an inexpensive, reliable, and objective RT test translatable to the training room or on the sidelines. The development of this study’s DTS protocol using inertial sensors during single and dual tasks within multiple conditions is the first step toward providing objective RT metrics using an accessible system at all levels of sports participation when a concussion is suspected or during return to play decisions. With this in mind, this study aimed to investigate if inertial sensors can be used in conjunction with the clinical DTS for RT assessments in out-of-laboratory environments. The validity and inter-rater reliability were assessed by comparing inertial sensors to the standard motion capture laboratory-test environment during multiple positions and with dual-task conditions. It was hypothesized that when performing the drop-stick test in sitting and standing positions and on different surfaces (standing on foam versus firm surface), both with and without dual-tasking, the drop-stick test with sensors would demonstrate good validity when compared with gold-standard motion capture systems in the laboratory.

## 2. Materials and Methods

### 2.1. Participants

Fourteen healthy adults (seven females and seven males) with a cohort mean (±standard deviation) age 24.7 (2.6) in years; height 173.4 (7.9) in cm; mass 75.3 (11.9) in kg; BMI 25.0 (3.8) participated. King-Devick’s scores were 40.0 (6.3) seconds. Participants were recruited using Chapman University IRB-approved flyers that were posted and distributed to the general public and around the campus at a local university. Participants were excluded if they had a recent (e.g., less than six months) musculoskeletal injury of lower body survey, had uncorrected visual problems or inner ear problems including dizziness and vertigo, had sustained a concussion within the past two months (e.g., 60 days), had received treatment for a respiratory infection within the last 30 days, had a diagnosed pulmonary disease or any other physical or systemic disorder or spinal deformities that may affect breathing, were color blind, and were pregnant or thought they might be pregnant.

### 2.2. Study Design

A mechanistic observational prospective cohort case-control study design was developed to assess the clinical drop-stick test utilizing an innovative protocol using single-and-dual-task conditions [30,31,32,33] to objectively evaluate the functional performance of reaction time and static postural control [34,35,36]. The purpose of the study was to develop DTS using inertial sensors and assess its reliability with three different conditions: (i) standing/sitting, (ii) single/dual tasking, and (iii) foam/firm surface standing. The purpose of testing DTS for validity and reliability among these conditions was to assess the applicability of this test using sensors (IMUs) in the clinical or field environment. The different tasks were selected to cover a heterogeneous level of use with varying levels of cognition and postural instability. The DTS protocol for condition and single and dual-task conditions were randomized for all subjects and all trials.

#### 2.2.1. Drop-Stick Test System

**Figure 1:**
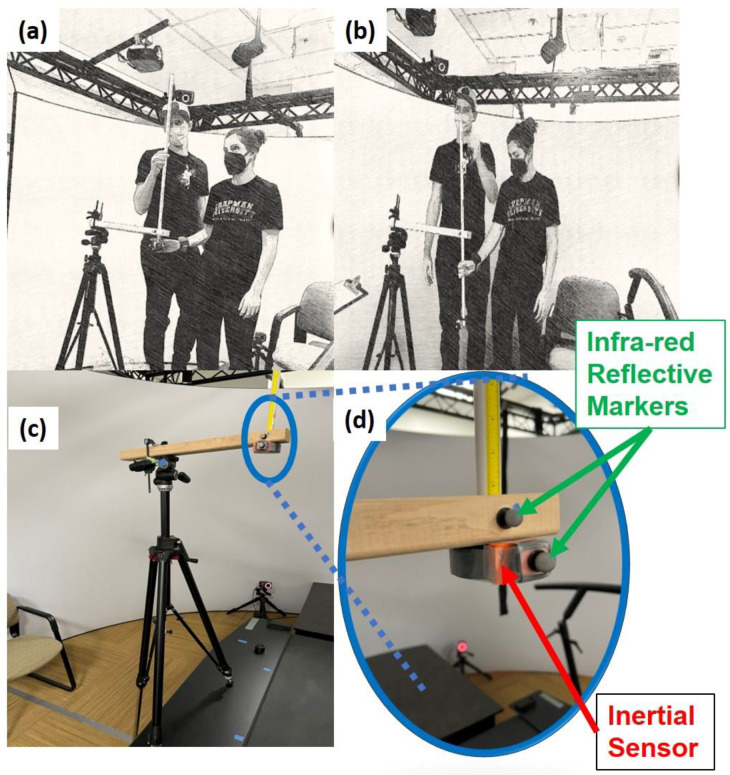
(**a**) Initial hand position before the drop, (**b**) a drop-stick catch, (**c**) the tripod-assisted initial position, (**d**) zoomed-out figure showing infrared markers and inertial sensor.

The drop-stick test system was developed similarly to previous studies to assess the clinical RT [25,26]. The experimental set-up involved participants standing on a force plate with the elbow flexing to approximately 90 degrees (Figure 1a). This drop-stick apparatus ensured the stick dropped in the same direction (i.e., vertical drop) for each attempt and was positioned to the side and adjusted to the level of the participant’s hand. The participant’s hand was open and placed without touching the stick’s weighted rubber disk portion (Figure 1a). Another researcher suspended the device vertically within the apparatus, which remained in the vertical plane (using a tripod for base support) with the participant’s open hand encircling the weighted rubber portion of the stick. The examiner dropped the stick using delays from two to five seconds which were random for each drop. The participant was instructed to catch the stick by closing the hand as fast as possible after the researcher released it (Figure 1b). Each participant was given one to two practice trials to become acquainted with the protocol and test before data collection. The clinical drop-stick RT device was a rigid stick 80 cm long, with a weighted rubber disk affixed to one end (Figure 1c). An inertial sensor and infra-red marker were affixed to the weighted rubber disk. Other infra-red reflective markers were placed at the wrist, on the inertial sensor affixed to the weighted rubber disk of the drop stick, and on the tripod support base (Figure 1d). Wireless sensor modules composed of Xsens MTw sensors packaged in a 47 mm × 30 mm × 13 mm plastic housing were used (Xsens Technologies BV, Enschede, The Netherlands). The sensors contain a 3D accelerometer [37,38] and 3D rate gyroscopes to measure acceleration and angular velocities. The sensors weighed 16 g, including the battery. The tri-axial accelerometer had a ±16 g capacity in the full range, and the gyroscope had ±2000°/s with a bandwidth of 3200 Hz, where g represents acceleration due to gravity (1g =9.8 m/s^2^). The accelerometer’s sensitivity was 31.2 LSB/g, and the gyroscope’s sensitivity was 14.375 LSB/s. Another sensor was affixed at the base of the wrist of the participant. The sampling frequency of the sensor and camera motion capture was set as 100 Hz.

#### 2.2.2. Dual Task Condition

**Figure 2:**
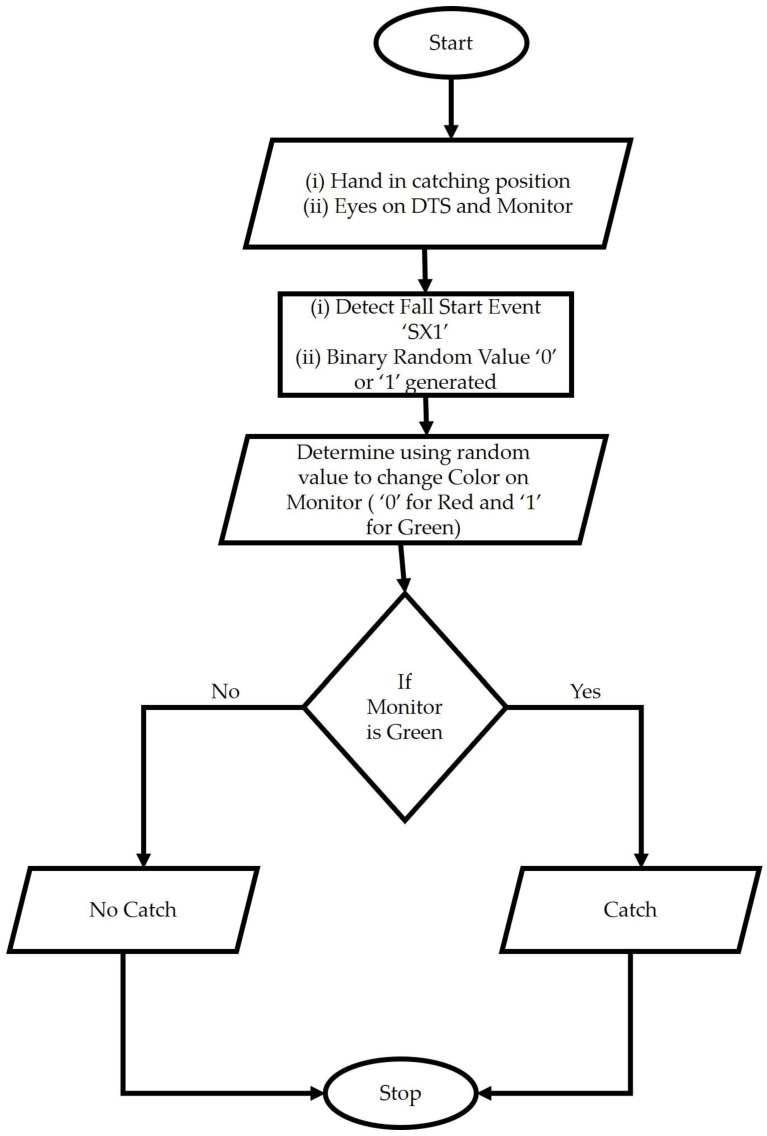
Schematic diagram showing how computer algorithm was created for dual-tasking condition.

In this condition, participants were asked to simultaneously monitor DTS and a computer monitor. A MATLAB (MATLAB (2022a), The MathWorks Inc., Natick, MA, USA) app was built, which could acquire real-time sensor data using Bluetooth and trigger a change in the color of GUI, either ‘green’ or ‘red.’ The algorithm to produce dual tasks on the computer monitor using the MATLAB app was provided, as shown by the flowchart in Figure 2. Participants were provided the cognitive task of identifying the color and making ‘catch’ (e.g., green) or ‘no catch’ (e.g., red) decisions accordingly.

### 2.3. Procedure and Protocol

Drop-stick test system (DTS)-based data collection was conducted in a single laboratory session lasting approximately 60 min. All participants who met the inclusion and exclusion criteria provided informed consent as approved by the Chapman University Institutional Review Board (IRB). After demographic data was collected (e.g., height, weight, BMI, hand and leg dominance), participants completed the King-Devick (K-D) [39,40] assessment. The K-D is a simple eye-scanning test commonly applied on the sidelines as an objective screen to evaluate if an athlete has sustained forces strong enough to result in a concussion. If positive, further objective testing in a non-field environment is warranted.

K-D is one of the most efficient and accurate sideline concussion screening tools with high sensitivity (0.86–1.00) and specificity (0.90–0.94) [41,42]. This assessment measures cognitive processing speed, rapid eye movement, and visual tracking, which, when impaired, correlate with suboptimal brain function [41,42]. Since the eyes tend to be the first identifiable sub-system when the brain is not optimally functioning, the K-D evaluates athletes by requiring eye-scanning of various numbers in different patterns, which the athlete must read aloud. The participant is timed in seconds; a good score is a faster score when compared with the athlete’s baseline score. Slower scores indicate less optimal brain function.

Once informed consent was obtained, and demographic data and K-D were collected, the participants entered the laboratory and were instrumented in preparation for the clinical stick-drop test. The researcher collecting the specific laboratory metric tests was blinded to the participants’ K-D scores.

### 2.3.1. Experimental Conditions

#### Single Task

The stick-drop test occurred under single-task testing conditions while standing and sitting on a force plate. A total of ten trials were collected under each single-task condition.

#### Dual Task

**Figure 3:**
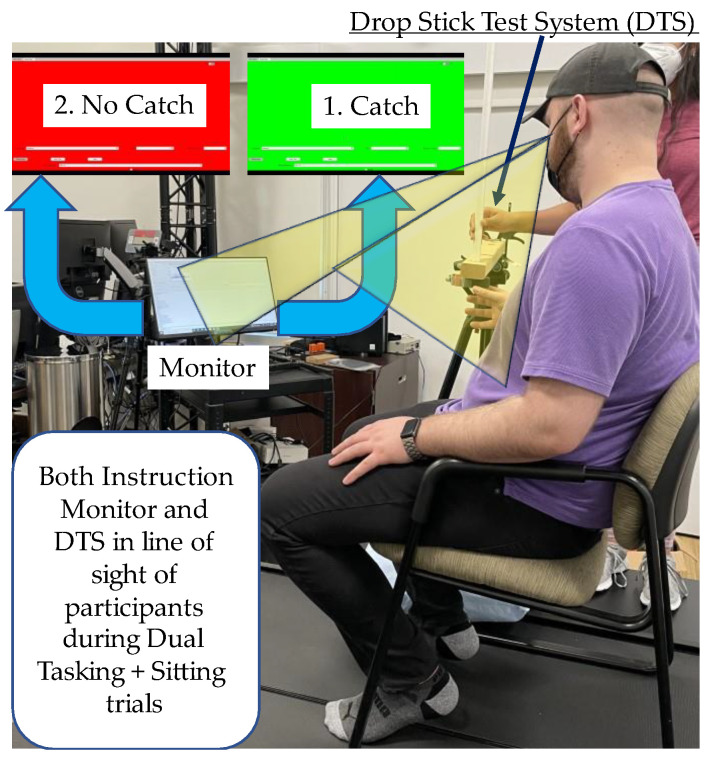
This picture shows the experimental setup for the dual-task condition. The participant sat and waited for instructions from the monitor, which randomly and instantaneously turned ‘green’ or ‘red’ after the drop. The participant was to ‘catch’ the stick if the monitor screen turned ‘green’ or let the stick fall (‘no catch’) if the screen turned ‘red.’.

During dual-task testing, participants visualized a computer screen placed 1.2 m in line of sight with their catch hand (Figure 3). They were instructed to catch the stick only when the monitor showed a green screen and not to catch it when it showed a red screen. Participants completed ten stick drops with five green and five red dual-task conditions in random order.

#### Foam Stand

Participants were instructed to stand on foam and perform catch during drop-stick tests.

#### No Foam Stand

Participants were instructed to stand on a firm surface during the drop-stick catch protocol testing.

#### Sit

Participant RTs were collected for the drop-stick test during the sitting condition. At least ten trials were collected for each situation. Furthermore, the drop-stick had measuring tape secured to it, allowing the researcher to manually read distance measures in inches converted to centimeters for analysis to compare to measurement data provided by motion capture and IMUs.

**Figure 4:**
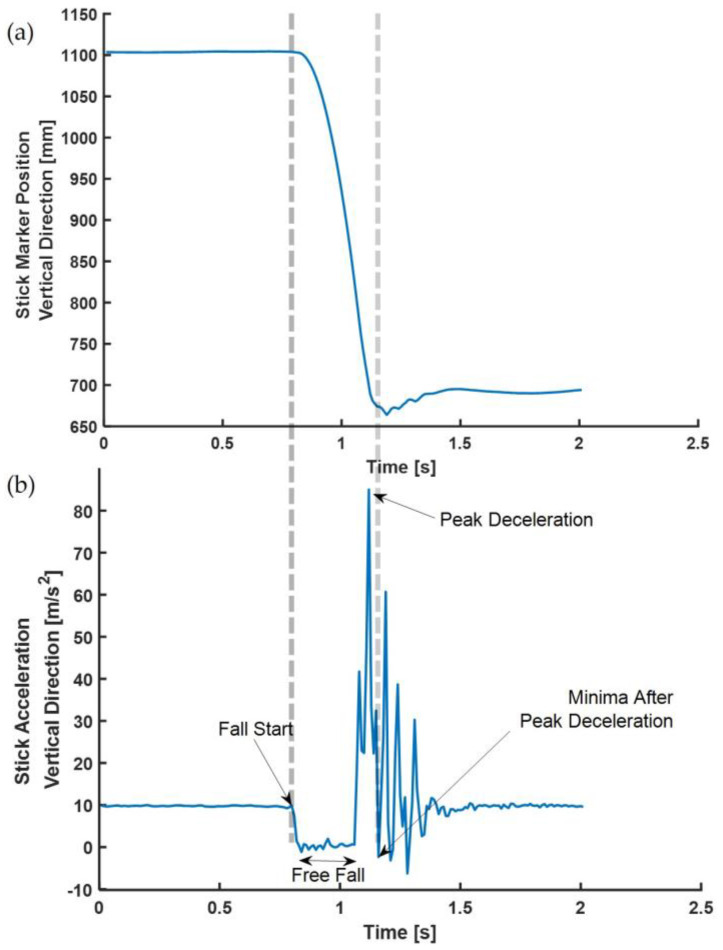
(**a**) Vertical marker position during a stick fall, (**b**) Vertical acceleration trajectory during a stick fall.

RT Evaluations: Data from the infra-red marker and sensor located on the base of the stick, as shown in Figure 1d, were used to evaluate RTs from all trials. The positional data for the vertical direction from the marker on the stick showed the trajectory during a stick fall (Figure 4a). The vertical acceleration measured by the stick during the fall is shown in Figure 4b. The RT was computed as the time between the dashed lines, as shown in Figure 4. The reaction times were evaluated using the stick’s infra-red marker and inertial sensor (Figure 4a,b). The MATLAB algorithms were developed to identify events automatically and were visually verified for each trial.

**Figure 5:**
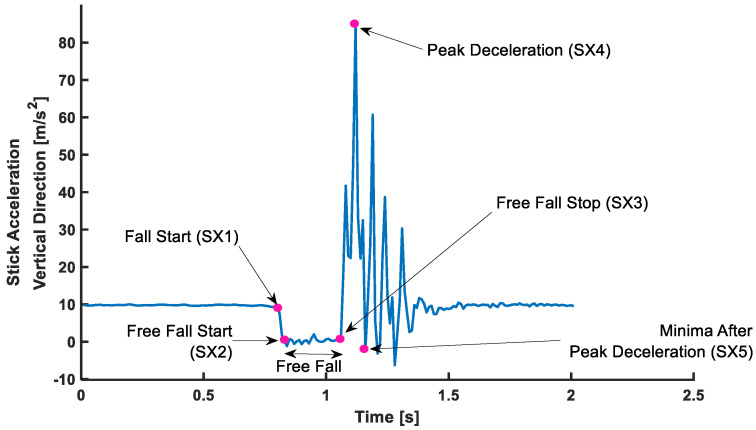
Five Stick fall events: (i) Fall start (SX1), (ii) Free Fall Start (SX2), (iii) Free Fall Stop (SX3), (iv) Peak Deceleration (SX4), (v) Minima after Peak Deceleration (SX5).

Inertial Sensor Event Identification: Five temporal events were identified from vertical acceleration from the sensor affixed to the drop stick (Figure 5). The sensor vertical acceleration identified (i) Fall Start (SX1) when the stick was released from the experimenter; (ii) Free fall, when the stick falls (SX2); (iii) Free fall stop, when the stick stops falling (e.g., caught) (SX3); (iv) peak deceleration (SX4); and (v) Minima after peak deceleration (SX5). These events were consistent in every stick fall; RT was evaluated as the time difference between SX5 and SX1.

Data Analysis and Statistics: The mean RT from each of the ten trials for every condition was used for data analysis. The mean RT of all participants was tested for normality using the Shapiro-Wilk test. Statistical significance was calculated at a 95% confidence level (*p* < 0.05). The inter-instrument reliability between DTS and camera-based motion capture systems was tested using intraclass correlation coefficient (ICC) assuming average fixed raters (e.g., ICC_3,1_), which measures inter-instrument reliability in terms of consistency [43]. ICC values of <0.5, 0.5–0.75, 0.75–0.9. and >0.90 indicate poor, moderate, good, and excellent reliability in the system. A low ICC value not only reflects the low degree of measurement agreement between the camera system and sensor system but also relates to a lack of variability among sampled subjects. Both consistency and absolute agreement are important since consistency concerns the degree to which a camera system (cs) can be equated to a sensor system (ss) plus a systematic error (c) (i.e., cs = ss + c). Whereas absolute agreement concerns the extent to which RT from the camera system (cs) equals RT from the sensor system (ss). This study calculated ICC estimates and their 95% confidence intervals using MedCalc Statistical Software version 19.2.6 (MedCalc Software Ltd., Ostend, Belgium) based on mean ratings (k = 3), absolute agreement, and a 2-way mixed-effects model. A Bland and Altman plot was used to evaluate the mean RT values measured from sensor and motion capture systems. The plot visualizes the differences in mean values of RT between the two devices against their means. A significantly different slope from zero suggests a proportional difference in RT between the sensor and motion capture, where the difference increases or decreases as the mean increases. The limits of agreement are calculated using a regression approach of the nonuniform differences [44,45]. All reliability data were analyzed in MedCalc Statistical Software version 19.2.6 (MedCalc Software Ltd., Ostend, Belgium). RTs were quantified by two measurement devices across all drop tests (Conditions and Tasks) to evaluate concurrent validity. The Pearson correlation coefficient was evaluated and described the magnitude of the associations between RT determined from the motion capture and the DTS (Table 1). The correlation values were categorized into three categories: poor (r ≤ 0.49), moderate (r = 0.50 to 0.74), and strong (r ≥0.75) [46].

## 3. Results

**Figure 6:**
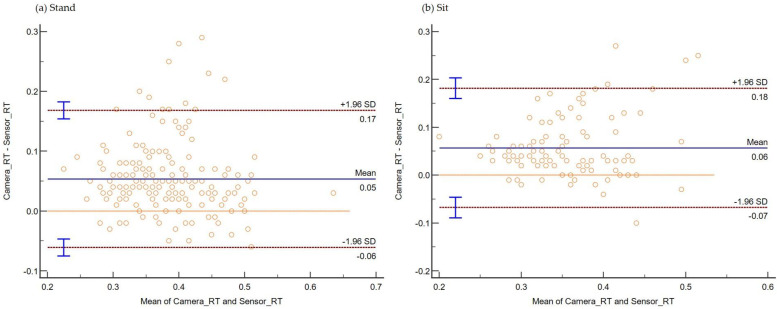
Plot differences between the camera and sensor systems RT versus the mean of two measurements during (**a**) standing and (**b**) sitting. The camera system reported a longer mean RT (bias) during standing by 0.05 s and sitting by 0.06 s.

**Figure 7:**
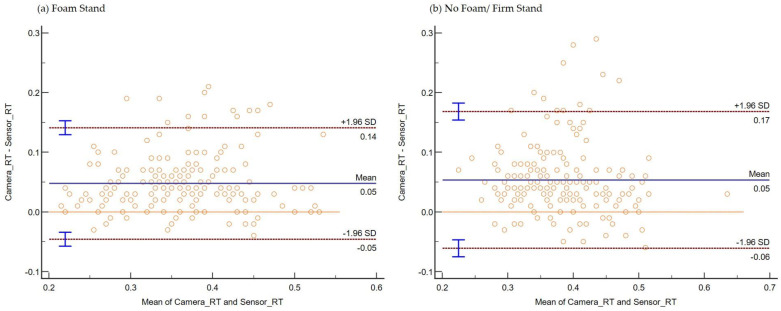
Plot differences between RT from the camera and sensor systems versus the mean of two measurements during (**a**) foam surface standing and (**b**) no firm surface standing. The camera system reported a longer mean RT (bias) during both conditions by 0.05 s.

**Figure 8:**
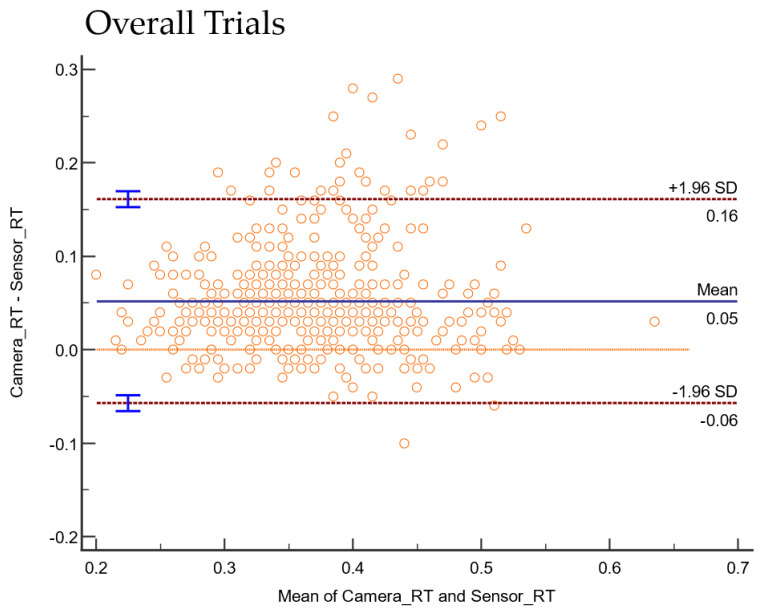
Plot differences between the camera and sensor systems RT versus the mean of two measurements for the overall trial with all conditions. Overall, camera systems were found to have a longer mean RT (bias) by 0.05 s.

**Figure 9:**
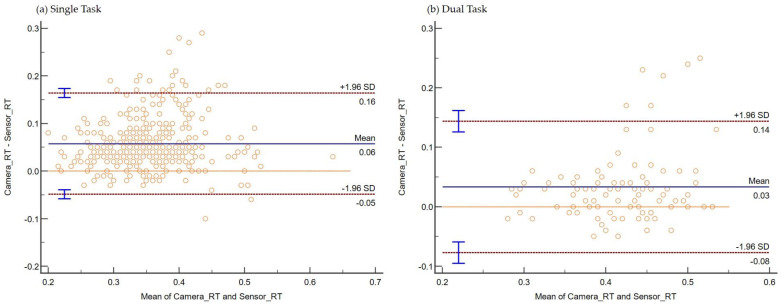
Plot differences between the camera and sensor systems RT versus the mean of two measurements during (**a**) Single Tasking and (**b**) Dual tasking. During the single task, the bias of RT was 0.06 s, and during dual tasking, the bias was 0.03 s.

The consistency and absolute agreement, as defined by ICC, are provided in Table 1 below. The Bland–Altman plot negating proportional differences and showing systematic bias in agreements is shown for (i) Stand (Figure 6a), (ii) Sit (Figure 6b), (iii) Standing on Foam (Figure 7a), (iv) Standing on a Firm surface (Figure 7b), (v) overall data points from all trials (Figure 8), (vi) Single Tasking (Figure 9a), and (v) Dual Tasking (Figure 9b) and the values are tabulated in Table 2. The temporal events during the drop-stick fall are shown in Figure 5. The duration of time between these events is tabulated in Table 3.

## 4. Discussion

A simple, quick, portable, cost-effective metric to assess RT is needed. The clinical stick-drop reaction time (RT) outcome measure is comparable to computer-based RT measures. It is both valid, with criterion and constructs validity, and reliable for measuring performance differences in concussed and non-concussed individuals, including under dual-task conditions [26,47,48]. This study was developed similarly to single condition and dual-task catch or no catch studies by Eckner and coworkers [25]. The drop-stick test system (DTS) developed for this study used an inertial sensor attached to a drop-stick to compare the reaction times from an inertial sensor to a motion capture system under differing conditions and both single and dual tasks.

The RT measurements from the fourteen participants using the camera system and sensor-based DTS showed moderate to good inter-instrument reliability with an overall ICC of 0.82 (95% CI 0.78–0.85) (Table 1). There was an overall dataset bias of 0.05 s from Bland–Altman plots depicting shorter RT by sensors compared with the camera system. This data provides the first evidence that the DTS is a reliable measurement tool. However, it has a bias of 50 ms compared with motion capture (Figure 6).

The DTS was validated for RT measurements for multiple conditions, including (i) standing, (ii) sitting, (iii) standing on foam, (iv) standing on a firm surface, (v) foam, and no foam standing. The DTS showed good consistency for standing with an ICC of 0.78 (95% CI 0.71–0.83), moderate consistency for sitting with an ICC of 0.70 (95% CI 0.56–0.80), good consistency for a single task with an ICC of 0.79 (95% CI 0.75–0.83), good consistency for dual tasking with an ICC of 0.75 (95% CI 0.64–0.83), very good consistency for standing on foam with an ICC of 0.88 (95% CI 0.84–0.91), and good consistency for standing on a firm surface with an ICC of 0.78 (95% CI 0.71–0.83) (Table 1).

Bland and Altman plots can quantify the agreement between two quantitative measurements by constructing limits of agreement. If the bias in the Bland–Altman plot is zero, the measurements obtained by the camera system or DTS gave the same results. But any measurement by instruments will have some degree of error, generating a variability of differences in the Bland–Altman plot. Most of the data points remained over the lower LOA. Thus, the camera system reported larger RTs compared with the DTS in standing (foam and firm surface), sitting, and single and dual-task conditions. This study’s temporal data differences between the camera and sensor systems are not unique. Previously reported temporal events of human movements such as sit-to-stand [49], sit-to-walk [50], and gait [21] have been shown to be comparable among the two motion capture systems (camera versus inertial sensors).

Although the camera-based motion capture is considered the gold standard for movement quantification, it requires an update to meet the evolving needs of on-site concussion assessment during play. With the recent COVID-19 pandemic, the assessment and rehabilitation paradigms are shifting to out-of-clinic environments. Platforms, such as the DTS, provide an objective measurement of functional RT while allowing the healthcare provider to assess concussion effects reliably over time. Moreover, integrating the DTS with a smartphone via wireless Bluetooth connectivity and automatic RT evaluations could provide quick assessment or comparative reports on the smartphone. The current study provided the first evidence to support this simple RT assessment using the DTS with inertial sensors (IMUs) under different conditions and during dual-tasking as a reliable measurement tool compared with the gold-standard motion capture.

The current study is innovative and provides a unique insight into the applicability of an RT testing protocol using inertial sensors under single and dual-task conditions, which are translatable into the training room. Lempke and coworkers demonstrated that the clinical stick drop and computer-based RT tests were comparable but suggested that the stick drop RT may be a better indicator when making a return to play decisions, especially under dual-task test conditions [48]. They also found that performance during stick drop RT was worse under dual-tasking, during which the cognitive load is increased, and as the complexity of the task was increased [48]. In this study, reliability was established between single and dual tasking. There was good consistency for a single task with an ICC of 0.79 (95% CI 0.75–0.83) and good consistency for dual tasking with an ICC of 0.75 (95% CI 0.64–0.83).

This study had several limitations, and the findings should be interpreted accordingly. The sample size was small and only reflected DTS reaction times within a young, healthy population. Since this study focused on the reliability of two measurement systems, gender was not selected as an independent factor. This may explain some of the outliers and variances within the results. However, subjects acted as their own control by performing the same tasks on both measurement systems. Previous research using visual stimuli RT assessments is mixed, with some findings supporting increased RT in males [51] and others finding females faster in a stick drop test [52]. Since our question was not focused on gender differences, this study was designed in line with Lynall and coworkers [53], who also compared multiple reaction-time tests, one of which was the drop-stick, but who did not specifically compare gender differences as the focus of that study was also on the reliability of different RT tests. Further testing of the DTS reliability on older adults is needed. In addition, our ICC values corroborate ICC values by Lynall and coworkers who reported ICC range from 0.77 to 0.87 [53]. Despite the variance and outliers, the DTS protocol can be considered a reliable measure using IMU sensors in out-of-laboratory environments. The next step will be to test the DTS protocol’s validity in populations with concussions to support its utility for measuring RT to provide an objective measure for sideline concussion assessment and in the training room during return to play decisions.

In recent years, concussions have been a growing issue, partly due to increased awareness and an improved understanding of the condition [54]. A concussion is a mild traumatic brain injury (mTBI) that often changes many neural processes. These changes may put those with concussions at risk for chronic neurologic deficits and increase the risk of musculoskeletal injuries [54]. Current data on methods for concussion diagnosis are limited, but the functional reaction time [54] is an essential biomarker for concussion assessment. Deficits in reaction time (RT) are common, post-concussion, and often persist once symptoms have abated [55,56,57]. American football has the highest rate of concussions among high school sports, with about 11 concussions occurring in 10,000 athletic exposures [58]. In games such as American football, a slower RT may place an athlete at risk for an increased number and severity of hits to the head [15,16,17,18]. Thus, field RT assessment is a crucial neurocognitive marker for recovery after a concussion. It is known that RT deficits may persist, decreasing sports performance post-concussion even when the athlete is cleared to return to play [48]. In this study, participants were recruited if they lacked a history of concussion and met all the exclusion and inclusion criteria. Furthermore, participants had a KD score of 40 ± 6.3, which is within the normal range of this demographic [59,60,61] to ensure sample homogeneity.

The availability of an inexpensive, portable, reliable, objective RT assessment performed under single and dual tasking is essential for creating safe athletic environments. RT can be measured at baseline pre-season testing, used for those suspected of sustaining a concussion, or for return-to-play decisions. Since RT reflects cognitive processing when performed under dual-task conditions, it may assess inhibitory function, which is vital to executive functions of the brain, especially for those who have suffered a concussive event [62]. To provide reliable RT measures, the tests must be consistent and have agreements with the existing gold standards (i.e., the camera system). Therefore, the standardization of the DTS protocol and the need for good DTS consistency is essential to track an individual’s change in RTs over time or collectively compare RTs at two different points to determine an intervention’s effect. This study’s sensor-based DTS system is reliable, valid, and affordable and can be readily adapted to varying on-field testing environments and set-ups. It is also easy to operate and requires minimal training and experience.

## 5. Conclusions

The findings of this study suggest that the DTS protocol and testing apparatus are reliable tools to measure RT and may be translatable to the field or training room for concussion assessment. Inertial sensors (IMUs) utilized under the protocol conditions during single and dual-task conditions strengthen claims for reliably measuring RT. These findings are promising for clinicians and researchers using RT to assess those suspected of experiencing a concussion and to make decisions regarding injury, recovery, and safe return to sports. The development of the DTS, which has the potential for accessible and reasonable measures for RT, increases the safety of environments at all levels of athletics. Future research will investigate DTS validity and reliability in clinical populations with mild TBI outside lab environments.

## Figures and Tables

**Table 1:** Intraclass correlation (ICC) analysis between the camera and sensor systems. Overall, results show moderate-to-good consistency and absolute agreement between the camera-based and sensor-based systems.

Condition	Consistency		Absolute Agreement			
	ICC	ICC Confidence Interval 95%	ICC	ICC Confidence Interval 95%	Datapoints	Pearson Correlation Coefficient
Overall	0.82	[0.78 to 0.85]	0.71	[0.14 to 0.86]	480	0.69
Standing	0.78	[0.71 to 0.83]	0.66	[0.10 to 0.84]	190	0.64
Sitting	0.70	[0.56 to 0.80]	0.57	[0.02 to 0.78]	100	0.55
Single Task	0.79	[0.75 to 0.83]	0.65	[−0.03 to 0.84]	370	0.66
Dual Task	0.75	[0.64 to 0.83]	0.69	[0.42 to 0.82]	110	0.61
Foam Standing	0.88	[0.84 to 0.91]	0.79	[0.18 to 0.91]	190	0.79
Firm Standing	0.78	[0.71 to 0.83]	0.66	[0.10 to 0.84]	190	0.64

**Table 2:** Bias, 95% Confidence Interval of Bias, Lower Limits of Agreement (LOA), 95% CI of lower LOA, Upper LOA, 95% CI of Upper LOA from Bland–Altman plots.

Condition	Bias	95% CI bias	Lower LOA	95% CI of Lower LOA	Upper LOA	95% CI of Upper LOA
Overall	0.05	[0.04 to 0.05]	−0.05	[−0.06 to −0.04]	0.16	[0.15 to 0.16]
Standing	0.05	[0.04 to 0.06]	−0.06	[−0.07 to −0.04]	0.16	[0.15 to 0.18]
Sitting	0.05	[0.04 to 0.06]	−0.06	[−0.08 to −0.04]	0.18	[0.16 to 0.20]
Single Task	0.05	[0.05 to 0.06]	−0.04	[−0.05 to −0.03]	0.16	[0.15 to 0.17]
Dual Task	0.03	[0.02 to 0.04]	−0.07	[−0.09 to −0.05]	0.14	[0.12 to 0.16]
Foam Standing	0.04	[0.04 to 0.05]	−0.04	[−0.05 to −0.03]	0.14	[0.12 to 0.15]
No Foam Standing	0.05	[0.04 to 0.06]	−0.06	[−0.07 to −0.04]	0.16	[0.15 to 0.18]

**Table 3:** Identification of drop stick events (SX1, SX2, SX3, SX4, SX5) during the fall and time interval between the events. The time interval between SX1-SX2, SX1-SX3, SX1-SX4, and SX1-SX5 are reported for single and dual tasks.

		Time Interval (SX1-SX2)			Time Interval (SX1-SX3)			Time Interval (SX1-SX4)			Time Interval (SX1-SX5)		
Condition	Task	Mean	SD	SE	Mean	SD	SE	Mean	SD	SE	Mean	SD	SE
Foam Stand	Dual Task	0.047	0.021	0.003	0.341	0.055	0.008	0.410	0.058	0.009	0.547	0.103	0.016
Foam Stand	Single Task	0.060	0.025	0.002	0.231	0.055	0.005	0.315	0.061	0.005	0.430	0.112	0.009
Sit	Dual Task	0.057	0.040	0.009	0.308	0.049	0.011	0.368	0.041	0.009	0.462	0.044	0.010
Sit	Single Task	0.057	0.030	0.003	0.233	0.052	0.006	0.317	0.061	0.007	0.404	0.083	0.009
Stand	Dual Task	0.057	0.032	0.005	0.332	0.065	0.009	0.404	0.060	0.008	0.535	0.098	0.014
Stand	Single Task	0.063	0.025	0.002	0.240	0.057	0.005	0.332	0.065	0.005	0.443	0.135	0.011

## Data Availability

The raw data supporting the conclusions of this article will be made available by the corresponding author upon reasonable request.
